# Risk factors for mortality in Stenotrophomonas maltophilia bacteraemia: a 22-year retrospective study in a Japanese acute care hospital

**DOI:** 10.1099/acmi.0.001194.v3

**Published:** 2026-08-10

**Authors:** Michiya Tanuma, Takayuki Sakurai, Hidemasa Nakaminami, Masayo Tanaka

**Affiliations:** 1 Department of Pharmacy, NTT Medical Center Tokyo, 5-9-22 Higashi-gotanda, Shinagawa-ku, Tokyo 141-8625, Japan; 2 Department of Clinical Microbiology, School of Pharmacy, Tokyo University of Pharmacy and Life Sciences, 1432-1 Horinouchi, Hachioji, Tokyo 192-0392, Japan; 3 Department of Infectious Diseases, NTT Medical Center Tokyo, 5-9-22 Higashi-gotanda, Shinagawa-ku, Tokyo 141-8625, Japan

**Keywords:** bacteraemia, risk factor, *Stenotrophomonas maltophilia*

## Abstract

*Stenotrophomonas maltophilia* is a Gram-negative bacterium associated with nosocomial infections and high mortality, although reported risk factors vary. We retrospectively analysed 43 cases of *S. maltophilia* bacteraemia at NTT Medical Center Tokyo (2002–2024). The overall mortality rate was 44.2%. Univariate analysis revealed that higher Sequential Organ Failure Assessment (SOFA) scores (*P*=0.010), central venous catheterization (CVC) (*P*=0.028), total parenteral nutrition (*P*=0.003) and diabetes mellitus (*P*=0.033) were significantly associated with mortality. Multivariate analysis suggested SOFA score (*P*=0.048) and CVC presence (*P*=0.039) as independent risk factors. Appropriate antimicrobial therapy was not significantly associated with reduced overall mortality. However, it was associated with improved survival in patients with SOFA scores ≥5 (*P*=0.029). Additionally, among patients with CVC, catheter removal was associated with improved survival (*P*=0.005). These findings suggest that the SOFA score and CVC were associated with mortality in *S. maltophilia* bacteraemia and indicate that the effectiveness of antimicrobial therapy may depend on disease severity. The study underscores the potential need to implement diverse infection-control strategies to prevent *S. maltophilia* transmission and to tailor antimicrobial therapy to disease severity.

## Data Summary

No supporting external data were generated for this work. The author has provided all supporting data within the article.

## Introduction

*Stenotrophomonas maltophilia* is a Gram-negative bacillus that causes various infections, including pneumonia and bloodstream infections. In hospital environments, *S. maltophilia* has been isolated from sources such as tap water, nebulizers, ventilator circuits and contaminated disinfectants. It is an opportunistic, biofilm-forming pathogen [1] that can colonize medical devices in hospitals and cause opportunistic nosocomial infections in specific patient groups with high morbidity and mortality rates.

Fluoroquinolones, tetracyclines and trimethoprim-sulfamethoxazole (TMP-SMX) are recommended for treating *S. maltophilia* infections [2]. However, empiric antimicrobial regimens commonly used for critically ill patients – most often *β*-lactams and glycopeptides – do not provide activity against *S. maltophilia* [3]. This can delay the start of appropriate therapy, which may be linked to mortality from *S. maltophilia* infections.

We previously investigated risk factors for *S. maltophilia* infection and reported high Sequential Organ Failure Assessment (SOFA) scores and central venous catheterization (CVC) as risk factors for infection [4]. However, the definitions of infection and colonization differ across prior studies, and our own investigation also noted the difficulty in accurately distinguishing between these conditions, identifying this issue as a study limitation. Accordingly, to identify risk factors associated with true *S. maltophilia* infections more accurately, it was necessary to restrict the analysis to cases in which *S. maltophilia* was isolated from sterile specimens, such as blood cultures. Furthermore, although a high mortality rate was observed in cases where *S. maltophilia* was isolated from sterile sites, the limited number of cases in this study made it difficult to evaluate risk factors for mortality [4].

Bacteraemia associated with *S. maltophilia* results in poor clinical outcomes [5, 6]. Reports on risk factors for mortality due to *S. maltophilia* infections vary across studies and include malignancy, implantation of an intravenous device, chronic respiratory disease, immunodeficiency, prolonged use of antimicrobial agents, septic shock, prolonged hospitalization and admission to an intensive care unit (ICU) [7–10]. Moreover, limited studies have investigated *S. maltophilia* bacteraemia; therefore, its characteristics relative to those of other types of Gram-negative bacteria, such as *Pseudomonas aeruginosa*, remain unclear [11]. The epidemiology of bacterial infections varies widely based on the characteristics of the medical institution and geographical location. Therefore, evaluating regional data on bacterial infections is essential for determining appropriate antimicrobial therapy strategies. However, cases of *S. maltophilia* bacteraemia are less frequent than those of other major Gram-negative pathogens in Japan [12], and the number of reports on *S. maltophilia* bacteraemia in Japan remains limited, leaving many clinical aspects insufficiently characterized [10, 13–15]. Therefore, in this study, we investigated *S. maltophilia* bacteraemia cases collected over 22 years to determine mortality rates and associated risk factors based on data from routine clinical practice. According to Japanese studies, the updated Charlson Comorbidity Index (CCI), intratracheal intubation, the SOFA score and ICU admission were significantly associated with increased mortality [14, 16]. However, findings regarding appropriate antimicrobial treatment varied. Furthermore, these studies did not address the mortality risk associated with each comorbid condition but rather focused on identifying risk factors associated with *S. maltophilia* bacteraemia in Japan. This study builds upon the findings of previous studies by examining not only the well-known risk factors but also those specific to various comorbid conditions. Additionally, to verify the effectiveness of appropriate antimicrobial therapy, we examined the mortality risk according to SOFA scores. Accumulating information in Japan will provide crucial data for establishing future treatments.

## Methods

### Study design, setting, and patients

**Fig. 1.**
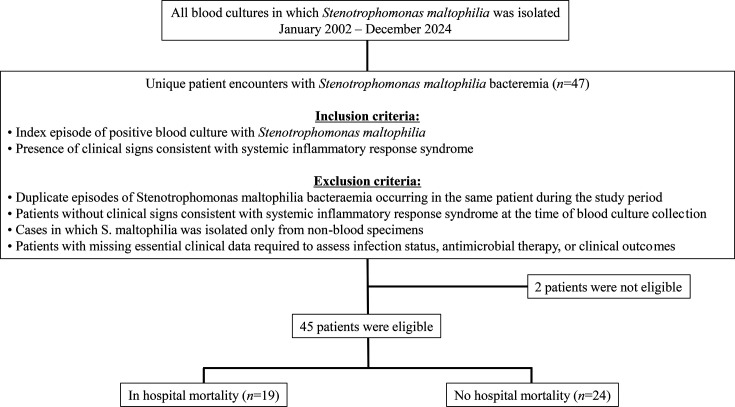
Flowchart of study enrolment for patients with *S. maltophilia* bacteraemia.

This retrospective study was conducted at the NTT Medical Center in Tokyo, Japan, an acute care hospital with 594 inpatient beds. Using a microbiology laboratory database, we identified cases of *S. maltophilia* detected in blood cultures at our hospital between January 2002 and December 2024 (Fig. 1). The March 2022–August 2023 window overlapped with the investigative period of our previous report [4]; in this study, we analysed newly obtained data in addition to three cases that were also included in the prior study. We defined the study period based on the maximum duration of electronic medical record data availability at our hospital. Additionally, we defined *S. maltophilia* bacteraemia as the presence of clinical signs of systemic inflammatory response syndrome (SIRS) [17] and at least one positive blood culture result. Although Sepsis-3 recommends the use of quick SOFA (qSOFA), previous reports have shown that qSOFA has higher specificity but lower sensitivity than the SIRS criteria [18]. Therefore, to minimize the risk of missing infectious cases, we adopted the SIRS criteria, which offer higher predictive sensitivity. In cases of multiple *S. maltophilia* cultures, only the first infectious episode was considered. Patients who did not exhibit signs of SIRS at the time of blood culture collection and those who did not have a positive blood culture result were excluded from the study. This study was approved by the Ethics Committee of NTT Medical Center Tokyo prior to its commencement (approval number: 000200023188-01). Owing to the retrospective and non-invasive nature of the study, the requirement for individual informed consent was waived. Information about the study and an opt-out option were available to patients on the hospital website. The study was conducted in accordance with the privacy policy guidelines to protect patients’ personal information.

### Potential risk factors and definitions

The primary endpoints were the 30-day mortality rate and the identification of risk factors for mortality. Cases with missing data were excluded from the analysis.

The onset of bacteraemia was defined as the date on which a positive blood culture was obtained. Polymicrobial bacteraemia was defined as the concomitant presence of bacteria other than *S. maltophilia*. The following variables were assessed as potential risk factors for death from *S. maltophilia* bacteraemia: sex, age, underlying disease (haematologic disease, solid malignancy, chronic obstructive pulmonary disease, diabetes mellitus, cerebrovascular disease, cardiovascular disease and chronic kidney disease), the SOFA score at the time of *S. maltophilia* isolation and ICU admission within 30 days prior to detection. History of surgery; the presence of devices, such as tracheal intubation, CVC, urinary tract catheter or stent; and hepatobiliary therapy (hepato-biliary-pancreatic drainage), such as percutaneous transhepatic gallbladder drainage, endoscopic biliary drainage or biliary stent, were also recorded. Comorbidities were selected based on those included in the CCI [19]. The source of bacteraemia was identified through the isolation of *S. maltophilia* in specimens obtained from other sites, including sputum, the biliary tract and the intestines, along with concurrent localized symptoms of active infection. In the absence of such findings, bacteraemia was classified as a primary infection [10]. Neutropenia was defined as an absolute neutrophil count <500 /μL. CVC removal was defined as catheter removal within 5 days of a positive blood culture result. The medication history of patients, including antimicrobials, antineoplastic agents, steroids and immunosuppressants, was recorded within 30 days of the detection of *S. maltophilia*. Antimicrobials continued for more than 3 days were considered previous drug exposure. Appropriate antibiotic therapy was defined as the use of at least one antibiotic with documented *in vitro S. maltophilia* susceptibility, such as TMP-SMX, minocycline (MINO) or fluoroquinolones, after the first blood sample collection. An appropriate empirical antibiotic was defined as the prescription of drug(s) that are effective against *S. maltophilia* isolates at an adequate dose within 72 h of blood culture collection. The dosage was adjusted according to renal function. To investigate the relationship between appropriate antimicrobial therapy and disease severity, a post hoc exploratory subgroup analysis was performed. This analysis involved the comparison of patients who had SOFA scores <5 with those who had SOFA scores ≥5, which is indicative of moderate to severe organ dysfunction and is strongly associated with increased mortality [3]. Septic shock was defined according to the Sepsis-3 criteria. Mortality was defined as death from any cause within 30 days of onset of bacteraemia.

The antimicrobial susceptibility of *S. maltophilia* was tested using the following agents: ceftazidime (CAZ), levofloxacin (LVFX), MINO and TMP-SMX.

### Blood culture and drug sensitivity test

All bacterial species were identified using conventional methods and/or a MALDI Biotyper (Bruker Daltonics, Billerica, MA, USA). Antimicrobial susceptibility was tested using the MicroScan Neg MIC3J (Beckman Coulter, Inc., CA, USA) and MicroScan WalkAway plus System (Beckman Coulter, Inc., CA, USA). The results were interpreted according to Clinical and Laboratory Standards Institute (CLSI) guidelines. Antimicrobial susceptibility was reassessed using previously determined minimum inhibitory concentration values, based on the breakpoints defined in CLSI breakpoints (M100-Ed33). The evaluation was independently performed by two infectious disease specialist pharmacists, and all assessments were in complete agreement.

### Statistical analysis

The *χ*^2^ test or Fisher’s exact test was used to determine risk factors for univariate comparisons between the two groups: death and survival. The Mann–Whitney *U* test was used to compare continuous variables. Logistic regression analysis was used to identify risk factors associated with death. For the logistic regression analysis, variables were selected based on clinical relevance and prior evidence. Because the number of events was limited, including all significant variables would have increased the risk of overfitting. Consequently, we employed a parsimonious set of predictors in accordance with the recommended event-per-variable criteria. The receiver-operating characteristic (ROC) curve was plotted, and the area under the curve (AUC) was calculated. Discrimination was evaluated using the ROC-AUC and its corresponding 95% confidence interval (CI). Calibration was assessed using the Hosmer–Lemeshow goodness-of-fit test. The association between appropriate antimicrobial therapy and 30-day mortality was assessed separately in patients with SOFA scores ≥5 and <5 using Fisher’s exact test. To evaluate whether the effect of appropriate therapy on mortality differed according to disease severity, the Mantel–Haenszel test for homogeneity was conducted. Survival rates for the CVC removal and non-removal groups were estimated using Kaplan–Meier curves and compared between groups using the log-rank test. Statistical significance was set at *P*<0.05. All statistical analyses were performed using SPSS version 24 (IBM Corp., Armonk, NY, USA).

## Results

### Clinical characteristics of patients with *S. maltophilia* bacteraemia

During the study period, 45 patients with *S. maltophilia* growth in their blood cultures were identified. Two of these patients were excluded from the study based on the exclusion criteria. A total of 43 patients with *S. maltophilia* bacteraemia were enrolled in this study and had a 30-day mortality rate of 44.2% (19/43 patients). Patient characteristics are shown in Table 1. The study cohort included 30 men and 13 women, with a median age of 75 years (interquartile range, IQR: 65.5–83 years). The median length of hospital stay before the onset of *S. maltophilia* bacteraemia was 33 days (IQR: 19.5–54.5 days). The most common comorbidities were haematologic diseases (16/43, 37.2%), followed by cardiovascular disease (14/43, 32.6%) and solid malignancy (12/43, 27.9%). The most common invasive procedure was total parenteral nutrition (TPN) (23/43, 53.5%), followed by urinary tract catheters (19/43, 44.2%) and CVC (17/43, 39.5%). The median SOFA score was 5 (IQR: 2.5–7). Only 11 cases (25.6%) had a definite source of bacteraemia, with the lungs (16.3%) being the most common source. Appropriate antimicrobial therapy was administered to 25/43 patients (58.1%): 48.1% received fluoroquinolones, 28.0% received MINO and 8.0% received TMP-SMX. Combination therapy was administered to 12.0% and 4.0% of patients who were treated with two and three drugs, respectively.

**Table 1.** Demographic and clinical characteristics associated with 30-day mortality

Risk factor	Previous study [4]	This study	Total	Survived	Death	OR (95% CI)	***P*-value**
	*n*=3	*n*=40	*n*=43 (%)	*n*=24 (%)	*n*=19 (%)		
Male, *n* (%)	0	30	30 (69.8)	15 (24)	15 (78.9)	–	0.244
Age [median, (IQR)]	84 (83.5–89)	72.5 (65–82)	75 (65.5–83)	77 (66.5–83.5)	69 (65–81)	–	0.372
SOFA score [median, (IQR)]	0 (0–3.5)	5 (3–7.25)	5 (2.5–7)	3 (1.75–6)	7 (4–8.5)	–	0.010
ICU admission (within 30 days)	0	6	6 (14.0)	3 (12.5)	3 (15.8)	2.08 (0.36, 11.89)	1
Neutropenia (<500 /μl)	0	14	14 (32.6)	6 (25.0)	8 (42.1)	5.09 (1.12, 23.14)	0.235
Surgery (within 30 days)	1	6	7 (16.3)	5 (20.8)	2 (10.5)	0.45 (0.08, 2.61)	0.437
Septic shock	0	10	10 (23.3)	4 (16.7)	6 (31.6)	2.31 (0.54, 9.79)	0.295
Intratracheal intubation	0	2	2 (4.7)	0 (0.0)	2 (10.5)	–	0.189
Total parenteral nutrition	1	22	23 (53.5)	8 (33.3)	15 (78.9)	7.51 (1.86, 30.16)	0.003
Urinary tract catheters	2	17	19 (44.2)	6 (25.0)	13 (68.4)	6.51 (1.71, 24.77)	0.004
Hepato-biliary-pancreatic drainage	1	9	10 (23.3)	6 (25.0)	4 (21.1)	0.80 (0.19, 3.37)	1
**CVC insertion**			17 (39.5)	6 (25.0)	11 (57.9)	4.13 (1.13, 15.10)	0.028
CVC removal/CVC insertion	1/2	5/15	6/17 (35.3)	5/6 (83.3)	1/11 (9.1)	0.02 (0.001, 0.39)	0.005
**Source of bacteraemia**							
Primary	2	30	32 (74.4)	18 (75.0)	14 (73.7)	0.93 (0.24, 3.70)	1
Lung	1	6	7 (16.3)	4 (16.7)	3 (15.8)	0.94 (0.18, 4.81)	1
Biliary tract area	0	2	2 (4.7)	1 (4.2)	1 (5.3)	1.23 (0.07, 21.86)	1
Intestinal	0	2	2 (4.7)	1 (4.2)	1 (5.3)	1.23 (0.07, 21.86)	1
**Comorbidities**							
Haematologic disease	1	15	16 (37.2)	7 (29.2)	9 (47.4)	2.19 (0.62, 7.70)	0.220
Solid malignancy	1	11	12 (27.9)	6 (25.0)	6 (31.6)	1.38 (0.36, 5.28)	0.633
COPD	0	1	1 (2.3)	0 (0.0)	1 (5.3)	–	0.442
Diabetes mellitus	1	6	7 (16.3)	1 (4.2)	6 (31.6)	10.62 (1.15, 98.10)	0.033
Cerebrovascular disease	1	5	6 (14.0)	4 (16.7)	2 (10.5)	0.59 (0.10, 3.62)	0.678
Cardiovascular disease	2	12	14 (32.6)	9 (37.5)	5 (26.3)	0.60 (0.16, 2.21)	0.437
Chronic kidney disease	1	4	5 (11.6)	4 (16.7)	1 (5.3)	0.28 (0.03, 2.72)	0.363
**Previous drug exposure**							
Steroid	0	19	19 (44.2)	7 (29.2)	12 (63.2)	4.16 (1.16, 15.00)	0.026
Antineoplastic agent	0	8	8 (18.6)	3 (12.5)	5 (26.3)	2.5 (0.51, 12.18)	0.432
Fluoroquinolone	0	14	14 (32.6)	9 (37.5)	5 (26.3)	0.60 (0.16, 2.21)	0.437
Carbapenem	1	26	27 (62.8)	11 (45.8)	16 (84.2)	6.30 (1.45, 27.46)	0.010
Fourth cephalosporin	0	9	9 (20.9)	3 (12.5)	6 (31.6)	3.23 (0.69, 15.21)	0.153
Anti-MRSA drugs	0	15	15 (34.9)	6 (25.0)	9 (47.4)	2.70 (0.74, 9.81)	0.126
Immunosuppressant	0	3	3 (7.0)	2 (8.3)	1 (5.3)	0.61 (0.05, 7.30)	1
**Length of hospital stay until bacteraemia onset [median, (IQR)]**	13 (9–17.5)	34 (20–58.25)	33 (19.5–54.5)	21 (11.8–37.5)	48 (26–76.5)	–	0.008
**Polymicrobial bacteraemia**	0	18	18 (41.9)	11 (45.8)	7 (36.8)	0.69 (0.20, 2.36)	0.553
**Appropriate antimicrobial therapy**	3	22	25 (58.1)	16 (66.7)	9 (47.4)	0.45 (0.13, 1.55)	0.203

COPD, chronic obstructive pulmonary disease; MRSA, methicillin-resistant *Staphylococcus aureus*.

### Risk factors associated with mortality due to *S. maltophilia* bacteraemia

**Fig. 2.**
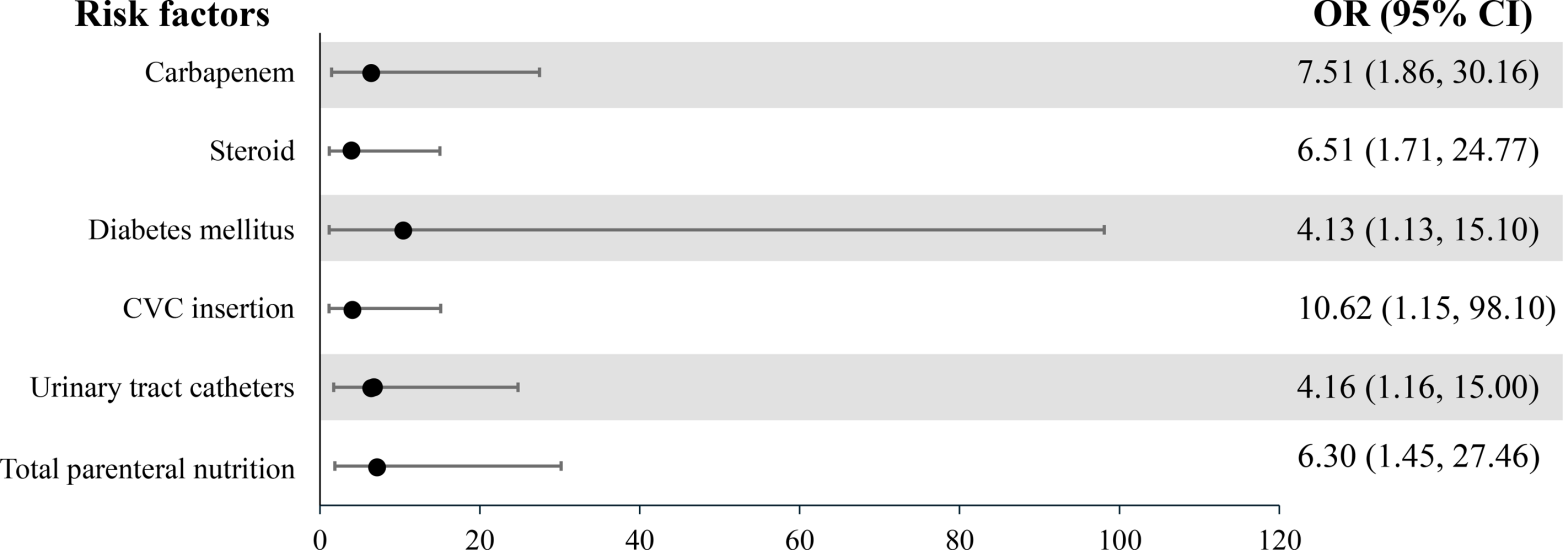
Risk factors associated with mortality in *S. maltophilia* bacteraemia.

In the univariate analysis, the following factors were significantly associated with mortality due to *S. maltophilia* bacteraemia (Fig. 2): SOFA scores (*P*=0.010), CVC (*P*=0.028), TPN (*P*=0.003), urinary tract catheters (*P*=0.004), steroid use (*P*=0.026), history of carbapenem use (*P*=0.010), length of hospital stay before *S. maltophilia* was detected (*P*=0.008) and diabetes mellitus (*P*=0.033). We performed a logistic regression analysis on the SOFA score, CVC insertion and administration of appropriate antimicrobial therapy (Table 2), all of which have been reported as risk factors for mortality. This analysis suggested the SOFA score (odds ratio, OR=1.262; 95% CI: 1.002–1.588; *P*=0.048) and CVC (OR=5.551; 95% CI: 1.094–28.184; *P*=0.039) as risk factors for mortality. Appropriate antimicrobial therapy was not found to be associated with 30-day mortality (OR=0.245; 95% CI, 0.048–1.255; *P*=0.091). The ROC curve analysis of our cohort yielded an AUC of 0.783 (95% CI: 0.634–0.931), indicating acceptable discrimination. We assessed model calibration using the Hosmer–Lemeshow goodness-of-fit test, which yielded a *P*-value of 0.598, suggesting an adequate model fit.

**Table 2.** Multivariate analysis of factors associated with 30-day mortality in patients with *S. maltophilia* bacteraemia

Factor	OR (95% CI)	***P*-value**
SOFA score	1.262 (1.002, 1.588)	0.048
Central venous catheterization	5.551 (1.094, 28.184)	0.039
Appropriate antimicrobial therapy	0.245 (0.048, 1.255)	0.091

The association between appropriate antimicrobial therapy and 30-day mortality was evaluated separately in patients with SOFA scores ≥5 and those with <5 using Fisher’s exact test. Among patients with SOFA scores ≥5, appropriate therapy was associated with a significantly lower 30-day mortality rate (35.7% vs. 88.9%; OR=0.07; 95% CI, 0.01–0.73; *P*=0.029). In contrast, among patients with SOFA scores <5, no significant difference in mortality was observed between those who received appropriate antimicrobial therapy and those who did not (36.4% vs. 22.2%; OR=2.00; 95% CI, 0.27–14.7; *P*=0.642). The Mantel–Haenszel analysis did not show a statistically significant association (OR, 0.42; 95% CI, 0.121–1.478; *P*=0.279) (Table 3).

**Table 3.** Effect of appropriate antimicrobial treatment on 30-day mortality by SOFA score severity group

Severity group	Appropriate antimicrobial therapy	30 day deaths (*n*)	Survivors (*n*)	Mortality rate (%)	OR (95% CI)	***P*-value**
SOFA score ≥5	No (*n*=9)	8	1	88.9	Reference	–
	Yes (*n*=14)	5	9	35.7	0.07(0.01–0.73)	0.029
SOFA score <5	No (*n*=9)	2	7	22.2	Reference	–
	Yes (*n*=11)	4	7	36.4	2.00(0.27–14.7)	0.642
**Mantel–Haenszel**	–	–	–	–	0.42(0.12–1.48)	0.279

**Fig. 3.**
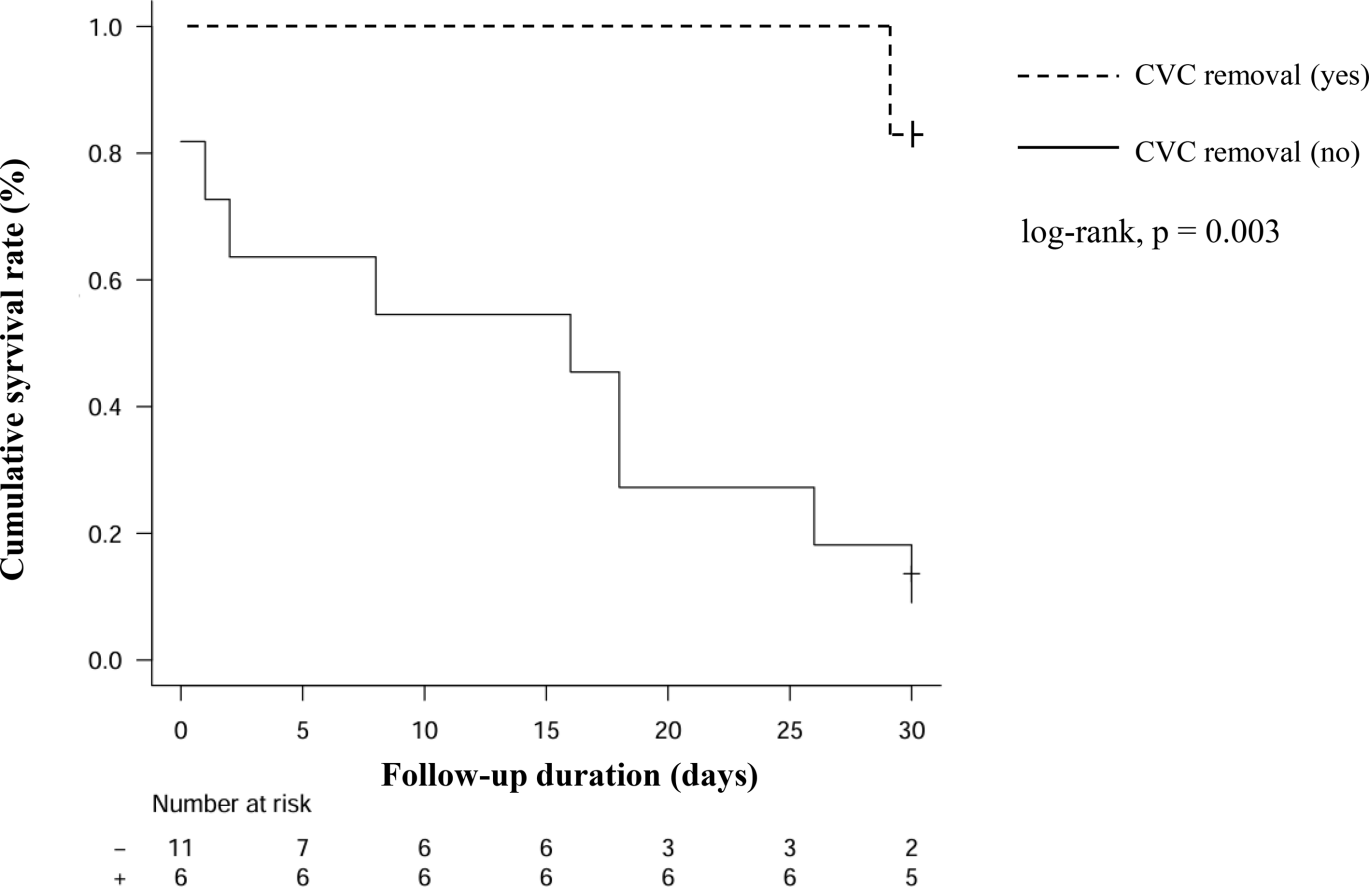
Kaplan–Meier survival curves for 30-day mortality according to central venous catheter removal in patients with *S. maltophilia* bacteraemia. Central venous catheterization not removed (solid line) and removed (dotted line); *P*<0.01.

A subgroup analysis was performed to compare the survival rates of patients with CVC implantation between the removal and non-removal groups (Fig. 3). According to the Kaplan–Meier curve, the 30-day survival rate was significantly higher in the CVC removal group (83.4%) than in the non-removal group (9.1%) (log-rank *P*=0.003).

### Drug susceptibility of *S. maltophilia* isolates and pathogens involved in polymicrobial bacteraemia

Drug susceptibility rates of *S. maltophilia* to CAZ, LVFX, MINO and TMP-SMX were 33.3%, 83.8%, 100% and 91.7%, respectively (Table 4). In this study, patients were given antibiotics for the treatment of *S. maltophilia* infections based on the results of antimicrobial susceptibility testing. Among patients with *S. maltophilia* bacteraemia, 18/43 (41.9%) had polymicrobial bacteraemia. The most frequently detected pathogens in polymicrobial bacteremia cases were *Enterococcus* spp. (33.3%), *Pseudomonas aeruginosa* (12.5%) and *Staphylococcus epidermidis* (12.5%). The mortality rates for polymicrobial bacteraemia and *S. maltophilia* bacteraemia alone were 38.9% (7/18) and 48.0% (12/25), respectively.

**Table 4.** Antibiotic susceptibility of *S. maltophilia* isolates

Antibiotics	*N*	Susceptible (%)	Intermediately susceptible (%)	Resistant (%)
Ceftazidime	33	33.3	9.1	57.6
Levofloxacin	37	83.8	5.4	10.8
Minocycline	36	100.0	0.0	0.0
Trimethoprim–sulfamethoxazole	36	91.7	0.0	8.3

## Discussion

This study was aimed at elucidating the mortality rate and risk factors associated with *S. maltophilia* bacteraemia based on data obtained from routine clinical practice. Our results revealed a mortality rate of 44.2% for patients with *S. maltophilia* bacteraemia. Additionally, higher SOFA scores and the presence of a CVC were independently associated with mortality. Notably, this is one of the few studies investigating risk factors for death due to *S. maltophilia* bacteraemia in acute care hospitals in Japan [10, 13, 14, 16]. However, the relatively small sample size of 43 cases collected over a 22-year period inherently limits the statistical power of our analyses, and the generalizability of the findings should be interpreted with caution.

In our previous study, we emphasized the SOFA score and CVC as key risk factors associated with *S. maltophilia* infection [4]. Although the study revealed a higher mortality rate in cases where *S. maltophilia* was detected in sterile specimens, a detailed analysis was not feasible. Therefore, in this study, we collected data on cases of bacteraemia over a 22-year period, in addition to incorporating three previously reported cases, and examined risk factors for mortality associated with *S. maltophilia* bacteraemia. We also investigated the relationship between CVC removal and mortality and further examined the association between appropriate antimicrobial therapy and mortality, for which previous reports have yielded differing results [14, 16].

The mortality rate of patients with *S. maltophilia* bacteraemia identified in this study (44.2%) is similar to rates reported in previous studies (29.6–65.1%) [20–23]. *S. maltophilia* possesses several intrinsic mechanisms that contribute to its broad antimicrobial resistance and the difficulty of clinical management. Most major resistance determinants are chromosomally encoded, and *β*-lactam resistance is primarily mediated by the L1 metallo-*β*-lactamase, which hydrolyses penicillins, cephalosporins and carbapenems, and the L2 serine *β*-lactamase, which hydrolyses aztreonam [24]. In addition, the organism exhibits resistance to multiple antimicrobial classes through the overexpression of multidrug efflux pumps and the production of aminoglycoside-modifying enzymes. Although carbapenems are used as empiric antimicrobial therapy for critically ill patients, *S. maltophilia* is intrinsically resistant, leading to a risk of delayed treatment. *S. maltophilia* also readily forms biofilms on intravascular catheters and other medical devices, promoting persistent colonization and reducing antimicrobial penetration. These combined mechanisms further limit effective therapeutic options, particularly in critically ill patients, and emphasize the importance of early identification of high-risk cases. Among the previously reported risk factors for mortality due to *S. maltophilia* infection [10, 20, 25, 26], biofilm formation is one of the most important pathogenic factors of *S. maltophilia* [27]. This ability enables *S. maltophilia* to adhere to the surfaces of medical devices, such as catheters. Accordingly, the presence of CVC, TPN and urinary tract catheters was associated with mortality in univariate analyses. In contrast, no significant difference was observed between tracheal intubation and hepato-biliary-pancreatic drainage. Reports of *S. maltophilia* detection in cholangitis are scarce, and the extent of its impact on mortality remains unclear. However, inadequate drainage in cholangitis is known to increase the risk of sepsis [28], and our findings may have been influenced by the degree of drainage achieved. Furthermore, invasive procedures such as endotracheal intubation have been reported as risk factors for *S. maltophilia* pneumonia [29]. Variations in infection control measures before and after intubation, which were not identified as risk factors in this study, may also have influenced these findings.

Additionally, in this study, diabetes mellitus was suggested as a possible risk factor for mortality. Although previous studies have reported a higher incidence of diabetes mellitus in the mortality group, only a few have found significant differences [21, 30]. Nevertheless, patients with diabetes mellitus are at a higher risk of developing common infectious diseases owing to impaired immune function, which may increase the risk of mortality [31]. In contrast, despite haematological diseases and neutropenia being associated with mortality, these conditions were not identified as risk factors in this study [25, 32, 33]. Studies investigating the risk of mortality among patients with *S. maltophilia* bacteraemia with haematological diseases reported that SOFA scores, age and pulmonary infection comorbidity are risk factors for mortality [34]. This suggests that blood disorders do not directly increase the risk of death; instead, their association with other factors influences mortality risk.

Furthermore, according to our results, no significant difference was observed in mortality between patients who received appropriate antimicrobial therapy and those who did not. This is consistent with the findings of recent studies, which reported that appropriate antimicrobial therapy did not have a significant effect on survival [26, 32]. These studies defined appropriate antimicrobial therapy as the use of at least one of the following antibiotics: TMP-SMX, MINO or fluoroquinolone. However, the Infectious Diseases Society of America guidance recommends combination antimicrobial treatment [2]. The optimal treatment for *S. maltophilia* remains undetermined. In clinical practice, monotherapy is more commonly used than combination therapy [35], and in the present study, combination regimens accounted for only 16% of all treatments. Although appropriate therapy did not show a significant impact on mortality in this cohort, it is possible that this finding was influenced by the overall intensity of antimicrobial treatment. Notably, the number of patients treated with combination therapy in our study was small; therefore, detailed evaluation of the therapy was not feasible. In this study, an association between appropriate antimicrobial therapy and reduced mortality was suggested in patients with SOFA scores ≥5, indicating that the effectiveness of appropriate antimicrobial therapy may vary depending on disease severity. In the subgroup analysis stratified by disease severity, appropriate antimicrobial therapy appeared to be associated with reduced mortality among patients with SOFA scores ≥5. These findings suggest that the effectiveness of appropriate antimicrobial therapy may vary according to the severity of illness. However, in the Mantel–Haenszel analysis adjusted for SOFA score-based severity, appropriate antimicrobial therapy showed a trend toward reduced mortality risk but did not reach statistical significance. This finding indicates that the treatment effect observed within severity strata may have been attenuated when the effects were averaged across the entire cohort. Accordingly, the interpretation of these findings should take into account the limited statistical power due to the small sample size. Previous reports from Japan showing no improvement in mortality with appropriate antimicrobial therapy included patients with relatively low SOFA scores: a median of 2 (95% CI, 1–3) in the survivor group and 4 (95% CI, 3–4) in the non-survivor group [16]. Conversely, reports showing improved mortality with appropriate antimicrobial therapy included cases among which 50% of patients had SOFA scores >6 [14]. Moreover, a study of ICU patients from Taiwan also demonstrated an association between appropriate antimicrobial therapy and improved survival [36]. Taken together, these findings suggest that, in patients with greater disease severity, *S. maltophilia* infection may have a greater impact on clinical outcomes, allowing the beneficial effects of appropriate antimicrobial therapy to become more apparent.

Additionally, significantly higher mortality was observed among patients with longer hospital stays and those who were administered carbapenems within the previous 30 days. These results are consistent with those of a previous study [37]. Patients with *S. maltophilia* bacteraemia often have a history of hospitalization due to other comorbidities, which result in longer hospital stays. Consequently, they are at a higher risk of opportunistic and nosocomial infections, potentially necessitating the use of broad-spectrum antimicrobial agents, such as carbapenems.

The logistic regression analysis in our study suggested that higher SOFA scores and the presence of a CVC were associated with an increased risk of mortality. These results are consistent with those of several previous studies [23, 26, 32]. Critically ill patients are suspected to be at an increased risk of *S. maltophilia* infection by CVC, which results in a significant direct mortality risk. Despite only 19 deaths in this study, the logistic regression model included three predictors, resulting in an events per variable (EPV) of 6.3. This is below the conventional threshold of EPV, which increases the risk of overfitting and may compromise model robustness and the stability of estimated associations. Therefore, caution is warranted when interpreting these results.

Moreover, the subgroup analysis of CVC implantation cases in the present study demonstrated markedly lower survival among patients in whom the CVC was not removed, a finding that aligns with previous reports. In interpreting the Kaplan–Meier comparison between the CVC removal and non-removal groups, it is essential to acknowledge that patients in the removal group must have survived sufficiently long to undergo the procedure. As a result, early deaths are unavoidably assigned to the non-removal group, thereby introducing the potential for immortal time bias. CVC removal is also more frequently undertaken in catheter-related infections and in clinically non-severe cases. Furthermore, even in severe cases in which CVC removal is considered desirable, the procedure may not be feasible due to the patient’s physiological instability. Consequently, the observed differences in survival may reflect not only the therapeutic effect of CVC removal but also confounding related to underlying disease severity. Nevertheless, prior studies have demonstrated that CVC removal confers a survival benefit even in cohorts without differences in SOFA or Acute Physiology and Chronic Health Evaluation II scores [32]. To resolve this issue, studies employing severity-based stratified analysis and propensity score matching are necessary. In this study, the number of cases was small, and the relationship between CVC removal and severity could not be examined. Additionally, patients with *S. maltophilia* bacteraemia in whom CVCs are not removed are known to experience recurrent bacteraemia [38]. Furthermore, previous studies have shown that CVC removal is effective in reducing mortality [20, 39]. Taken together, these findings indicate that catheter removal is associated with improved survival; however, this association warrants cautious interpretation within the clinical context.

Although recent studies have demonstrated increased resistance rates to TMP-SMX and MINO, as well as the emergence of strains resistant to these two drugs [40–42], previous studies in Japan reported resistance rates of 1.4–17.7% to TMP-SMX and 0% to MINO [15, 43, 44]. However, we did not observe any two-drug-resistant strains, and susceptibility to MINO and TMP-SMX was maintained. Our findings suggest that these drugs are currently adequate treatment options. This study was a long-term research project during which revisions were made to the CLSI breakpoints. We reevaluated susceptibility based on historical results using the CLSI M100-Ed33 criteria. When interpreting the results of this study, it is crucial to note that the current breakpoint criteria (CLSI M100-Ed35) for MINO are more stringent.

In this study, polymicrobial bacteraemia did not impact the mortality rate of patients with *S. maltophilia* bacteraemia, given the higher rate among patients with *S. maltophilia* alone. For example, *Enterococcus* spp. were the most frequently identified organisms in this study (33.3%), and their simultaneous presence is a risk factor for mortality [10]. However, the mortality rate of patients with polymicrobial bacteraemia was not higher than that of patients with *S. maltophilia* infection alone [21, 45]. This difference in mortality associated with microbial infection status may be due to the underlying host characteristics of the infected population rather than the effect of *S. maltophilia* infection itself.

Despite the notable results, this study had several limitations. First, this was a single-centre study. Accordingly, the characteristics of the patients with *S. maltophilia* bacteraemia were limited. Further studies are required to generalize the risk factors of *S. maltophilia* bacteraemia to the Japanese population. Second, owing to the small sample size, several risk factors may not have reached statistical significance. In addition, the limited sample size resulted in wide confidence intervals, and these estimates should be interpreted with caution. Furthermore, because the number of events was limited, several potential confounders – including ICU admission, prior antibiotic exposure and length of hospital stay – could not be fully adjusted for in the multivariate analysis, and residual confounding by these factors cannot be excluded. Third, although a strong association between CVC removal and improved survival was observed, these findings must be interpreted carefully. CVC removal is more likely to be performed in non-severe cases, whereas the removal may not be feasible in critically ill patients [46]. As a result, confounding by disease severity and immortal time bias may have influenced the observed survival differences. Thus, causal inferences regarding the effect of CVC removal should be made with caution. Thus, confounding by this factor cannot be ruled out. Therefore, caution should be exercised when interpreting the results. Another limitation is the use of SIRS as the criterion for defining *S. maltophilia* bacteraemia. This approach may have resulted in the inclusion of cases that were not true infections. Although SIRS was adopted to prioritize sensitivity and reduce the likelihood of missed infectious episodes, it is not consistent with the Sepsis-3 framework, which recommends qSOFA. Because SIRS has lower specificity than qSOFA, the possibility that non-infectious systemic inflammation was misclassified as infection-associated SIRS cannot be excluded. This difference in assessment criteria should be taken into account when interpreting the results of this study. Finally, determining the cause of death was difficult, and the effect of comorbidities on mortality was unclear. Therefore, we believe that future multicentre prospective cohort studies will yield clearer results.

In conclusion, we identified mortality risk factors for *S. maltophilia* bacteraemia in Japanese acute care hospitals. Patients with *S. maltophilia* bacteraemia had a high mortality rate, and SOFA scores and CVC use were identified as risk factors for mortality. Meanwhile, appropriate antimicrobial therapy was not found to have a significant association with mortality. However, among patients with SOFA scores ≥5, a signal towards improved survival was observed. Additionally, although removing CVCs may reduce the risk of mortality, multicentre, prospective cohort studies are required to draw definitive conclusions. In the present study, an association between appropriate antimicrobial therapy and reduced 30-day mortality was suggested among patients with a SOFA score of ≥5. However, the test for interaction using the Mantel–Haenszel method was not statistically significant, and both the sample size and the number of events were limited; therefore, any difference in treatment effect according to disease severity should be interpreted as a hypothesis-generating finding.

## Abbreviations

AUC: area under the curve
CAZ: ceftazidime
CCI: Charlson Comorbidity Index
CI: confidence interval
CVC: central venous catheterization
EPV: events per variable
ICU: intensive care unit
IQR: interquartile range
LVFX: levofloxacin
MINO: minocycline
OR: odds ratio
qSOFA: quick SOFA
ROC: receiver-operating characteristic
SIRS: systemic inflammatory response syndrome
SOFA: Sequential Organ Failure Assessment
TMP-SMX: trimethoprim-sulfamethoxazole
TPN: total parenteral nutrition
